# 3,3′-Diindolylmethane Induces G_1_ Arrest and Apoptosis in Human Acute T-Cell Lymphoblastic Leukemia Cells

**DOI:** 10.1371/journal.pone.0034975

**Published:** 2012-04-13

**Authors:** Lyndsey E. Shorey, Amanda M. Hagman, David E. Williams, Emily Ho, Roderick H. Dashwood, Abby D. Benninghoff

**Affiliations:** 1 Department of Environmental and Molecular Toxicology, Oregon State University, Corvallis, Oregon, United States of America; 2 Linus Pauling Institute, Oregon State University, Corvallis, Oregon, United States of America; 3 Department of Animal, Dairy and Veterinary Sciences, Utah State University, Logan, Utah, United States of America; 4 Department of Nutrition and Exercise Sciences, Oregon State University, Corvallis, Oregon, United States of America; 5 School of Veterinary Medicine, Utah State University, Logan, Utah, United States of America; 6 The Utah Science Technology and Research Applied Nutrition Research, Utah State University, Logan, Utah, United States of America; University of Chicago, United States of America

## Abstract

Certain bioactive food components, including indole-3-carbinol (I3C) and 3,3′-diindolylmethane (DIM) from cruciferous vegetables, have been shown to target cellular pathways regulating carcinogenesis. Previously, our laboratory showed that dietary I3C is an effective transplacental chemopreventive agent in a dibenzo[*def,p*]chrysene (DBC)-dependent model of murine T-cell lymphoblastic lymphoma. The primary objective of the present study was to extend our chemoprevention studies in mice to an analogous human neoplasm in cell culture. Therefore, we tested the hypothesis that I3C or DIM may be chemotherapeutic in human T-cell acute lymphoblastic leukemia (T-ALL) cells. Treatment of the T-ALL cell lines CCRF-CEM, CCRF-HSB2, SUP-T1 and Jurkat with DIM *in vitro* significantly reduced cell proliferation and viability at concentrations 8- to 25-fold lower than the parent compound I3C. DIM (7.5 µM) arrested CEM and HSB2 cells at the G_1_ phase of the cell cycle and 15 µM DIM significantly increased the percentage of apoptotic cells in all T-ALL lines. In CEM cells, DIM reduced protein expression of cyclin dependent kinases 4 and 6 (CDK4, CDK6) and D-type cyclin 3 (CCND3); DIM also significantly altered expression of eight transcripts related to human apoptosis (*BCL2L10*, *CD40LG*, *HRK*, *TNF*, *TNFRSF1A*, *TNFRSF25*, *TNFSF8*, *TRAF4*). Similar anticancer effects of DIM were observed *in vivo*. Dietary exposure to 100 ppm DIM significantly decreased the rate of growth of human CEM xenografts in immunodeficient SCID mice, reduced final tumor size by 44% and increased the apoptotic index compared to control-fed mice. Taken together, our results demonstrate a potential for therapeutic application of DIM in T-ALL.

## Introduction

Acute lymphoblastic leukemia (ALL), the most frequently diagnosed cancer in children ages 0 to 19 years [1], comprises a diverse population of malignant lymphoid progenitors undergoing clonal proliferation at various stages of differentiation [2]. The American Cancer Society estimates that 6,050 people in the United States will be diagnosed with ALL in 2012 [3], and incidence rates of ALL have increased significantly over the past thirty years [1]. Cases of T-cell origin (T-ALL) comprise 15% of ALL patients. Prognosis for these patients is poor, because they are less responsive to combination chemotherapy and are more likely to relapse than their B-cell counterparts [4].

New strategies in cancer therapy utilize drugs that specifically target aberrant signaling pathways in order to reduce toxic side effects, yet such specific therapies are only effective in a small percentage of this highly heterogeneous disease population. For example, more than half of T-ALL cases are characterized by a gain-of-function mutation in the Notch1 receptor, which leads to constitutive activation of Notch-mediated cell proliferation and survival [5], [6], [7]. Therapeutic gamma secretase inhibitors (GSIs) prevent cleavage of the intracellular Notch (ICN) domain and subsequent transcriptional activation of Notch target genes [8], [9], [10]. Subpopulations of T-ALL patients and cell lines (including CEM, SUP-T1, and Jurkat cells) are insensitive to GSI therapy, presumably due to mutations that result in constitutive ICN expression or additional mutations in genes downstream [8] such as phosphatase and tensin homolog (PTEN), a tumor suppressor and negative regulator of the PI3K/AKT/mTOR signaling pathway [11]. Due to the complexity and diversity of T-ALL signaling pathways, therapeutic efficacy and safety may be improved through the use of natural products that target multiple cancer signaling pathways, either alone or adjuvant to systemic or directed chemotherapy [12], [13].

Evidence from epidemiological and animal studies shows that modification of the diet to increase consumption of cruciferous vegetables is sufficient to reduce cancer risk (reviewed in [14], [15]). Furthermore, maternal consumption of vegetables was shown to be inversely associated with ALL in a population-based study [16]. The bioactive food component indole-3-carbinol (I3C) is produced from the hydrolysis of glucobrassicin, which is present at high concentrations in cruciferous vegetables, such as Brussels sprouts, broccoli, cabbage and cauliflower [17]. The anticancer effects of I3C have been well-documented in various tumor cell types including colon, breast, and prostate (reviewed in [18], [19]). However, I3C is unstable in acidic environments such as the stomach and rapidly undergoes self-dimerization and oligomerization to yield over 15 acid-condensation products (ACPs) [20]. A major product of this reaction *in vitro* [21] and *in vivo* [22] is 3,3′-diindolylmethane (DIM). Because DIM has greater stability than the parent compound [20], [23], [24], it is expected that DIM contributes significantly to the anticancer effects of dietary I3C and is more effective at an equivalent molar dose.

Herein, we report for the first time that DIM markedly reduces the proliferation and survival of four different human T-ALL cell lines, which were selected to represent the heterogeneity of the disease. The anticancer effects of DIM were exerted by modification of critical regulators in the cell cycle pathway leading to induction of G_1_ arrest and apoptosis. Subtle differences in sensitivity within this group of cell lines were observed, although DIM was more potent than its parent compound I3C in all cases. Of particular importance was the observation that DIM reduced growth of human CEM cells in a xenograft model when supplemented through the diet. Collectively, the data presented below suggest that DIM could be an effective anticancer agent in T-ALL cases originating from T cells at different stages of differentiation and at concentrations that can be reasonably achieved *in vivo* in humans and in animal models.

## Materials and Methods

### Materials

The following chemicals and reagents were purchased from the indicated suppliers: I3C from Sigma-Aldrich Co. (St. Louis, MO), Matrigel Matrix from BD Biosciences (Franklin Lakes, NJ) and ViaCount Flex Reagent from Millipore (Billerica, MA). DNase I and the NuPAGE system for SDS-PAGE, including 10% and 4–12% Bis-Tris gels and appropriate electrophoresis and transfer buffers, were purchased from Invitrogen (Carlsbad, CA). Antibodies for immunoblotting were obtained from Cell Signaling Technology (Danvers, MA), including β-actin and α-tubulin primary antibodies and the Cell-Cycle Regulation Antibody Sampler Kit (contains primary antibodies for CCND3, CDK4, and CDK6 as well as HRP-linked anti-mouse and anti-rabbit IgG secondary antibodies). DIM was kindly provided in a bioavailable formula (BioResponse-DIM, herein referred to as DIM) by BioResponse, LCC (Boulder, CO), which was certified to contain 30% DIM (wt/wt) by Eurofins-Alpha Laboratories (Petaluma, CA). This bioavailable form of DIM, rather than the pure crystalline DIM, has been utilized for many of the preclinical and clinical studies in the published literature and is the common form provided in commercial dietary supplements. For these reasons, we selected the BioResponse formula for the experiments outlined below. Experimental concentrations reported in this study were adjusted accordingly (*e.g.* treatment with 12.3 µg/ml BioResponse DIM is equivalent to 3.7 µg/ml DIM, or 15 µM DIM).

### *In vitro* experiments with human CEM cells

#### Cell lines and culture conditions

T-ALL is a heterogeneous disease resulting from the developmental arrest and abnormal proliferation of T-cells at different stages of maturation [25]. Four human T-ALL lines representing this heterogeneity were selected for this study, including human CCRF-CEM (CEM) cells, CCRF-HSB2 (HSB2) cells, SUP-T1 cells and Jurkat cells (see Table 1). Cell lines were characterized by their respective vendors at time of accessioning, and cells were passaged fewer than 15 times and no longer than 3 months after acquisition. All cell lines were maintained in phenol red-free RPMI-1640 medium (Sigma-Aldrich) containing 10% (v/v) charcoal-stripped, heat-inactivated fetal bovine serum (FBS; Atlas Biologicals, Fort Collins, CO or Caisson Laboratories, Logan, UT) in a humidified incubator at 37°C with 5% CO_2_. The CEM line was selected for further characterization and study in a xenograft model based on its classification as an immature lymphoblastic T-cell population with an immunophenotype similar to that observed in our murine model of transplacental carcinogenesis [26] and its demonstrated ability to form solid tumors in subcutaneous xenograft models [27]. DIM and I3C were prepared as concentrated stock solutions in DMSO, which were stored at −80°C protected from light. For *in vitro* experiments, cells were seeded 24 hr prior to treatment at appropriate concentrations for each specific endpoint. On the day of treatment, dilutions of DIM and I3C were prepared so that all experimental treatments contained 0.1% DMSO (v/v), including a vehicle control.

**Table 1:** Human T-ALL cell lines used in this study.

Cell line (abbreviation)	CCRF-HSB2 (HSB2)	CCRF-CEM (CEM)	SUP-T1	Jurkat (JM)	Reference
**Source (Cat #)** *	NIH-AIDS (497)	ATCC (CCL-119)	NIH-AIDS (100)	NIH-AIDS (4668)	
**Age (years)/sex**	11/m	3/f	8/m	14/m	[25]
**Immunophenotypic classification** a	Pre-T	Pre-T	Cortical T	Mature T	[25]
CD3 (cytoplasmic)	−	+	+	+	[25]
CD3 (surface)	−	−	−	+	[25]
CD4	−	+	+	+	[25]
CD8	−	−	+	−	[25]
CD1a	−	−	+	−	[25]
**Somatic mutations in T-ALL tumor suppressors** a					
CDKN2A(p16)	+	+	+	+	[47]
RB1	−	−	−	−	[47]
TP53	−	+	+	+	[47]
PTEN	−	+	−	+	[47]
**Oncogene profile (frequency in T-ALL)**					
NOTCH1 activating mutation (50–60%)^a^	+	+	+	+	[47], [53]
TAL1 expression (25%)^b^	+++	++	−	++	[53]
STIL-TAL1 fusion^a^	+	+	−	−	[53]
LYH1 expression^b^	+	++	−	−	[53]

* ATCC, American Type Tissue Collection; NIH AIDS Research and Reference Reagent Program.

a Indicates presence (+) or absence (−).

b Indicates relative expression (−, +,++, or +++).

#### Cell proliferation, viability and apoptosis

T-ALL cells were treated with 0 up to 60 µM DIM or 0 up to 500 µM I3C for up to 48 hr. The concentration of viable cells was determined at each indicated time point by the ViaCount Assay (Millipore, Billerica, MA) as recommended by the manufacturer using either the Guava Personal Cell Analyzer (Guava Technologies, Inc., Hayward, CA) or the Accuri C6 flow cytometer (BD Accuri Cytometers, Inc., Ann Arbor, MI); assay performance was comparable on both instruments. Raw data were compared to the time-zero control for cell proliferation and the time-matched control for viability. Concentration values for 50% inhibition (IC_50_) of T-ALL cell proliferation and viability by I3C and DIM were calculated by non-linear regression using a sigmoidal dose-response with variable slope (Prism 5, GraphPad Software, La Jolla, CA).

#### Cell-cycle analysis

T-ALL cells were treated with 0 to 15 µM DIM for up to 48 hr, rinsed in cold PBS, fixed in ice cold 70% EtOH, and stored at least overnight at −20°C. On the day of analysis, cells were washed with PBS and incubated for 30 min in the dark in staining solution (25 µg/ml propidium iodide, 0.1% (v/v) Trition X-100 and 0.2 mg/ml RNase in PBS). Flow cytometry was used to determine cellular DNA distribution using the Guava PCA or Accuri C6 instruments and the number of cells in each cycle were analyzed using MultiCycle software (Phoenix Flow System, San Diego, CA) or FlowJo Cytometry Analysis Software (Ashland, OR).

#### Immunoblotting

Cells were treated with 0 to 15 µM DIM for 12 or 24 hr or with 0 to 500 µM I3C for 24 hr, then lysed in IP lysis buffer (20 mM Tris, 150 mM NaCl, 1 mM EDTA, 1 mM EGTA, 1% (v/v) Triton X-100, 2.5 mM Na_4_P_2_O_7_·10H_2_O, 1 mM C_3_H_9_O_6_P, 1 mM Na_3_VO_4_, 1 µg/ml leupeptin and 0.5% protease inhibitor cocktail III (EMD Chemicals, Gibbstown, NJ)). Protein concentration was determined using the Coomasie Plus Assay (Thermo Scientific, Rockford, IL) and an equal amount of protein for each sample was separated by SDS-page electrophoresis and transferred to nitrocellulose membranes. Membranes were blocked for 1 hr in 5% non-fat milk or BSA prior to overnight incubation at 4°C with primary antibodies for CCND3, CDK4, or CDK6 (all 1∶1000 dilution). Membranes were subsequently incubated with the appropriate HRP-conjugated secondary antibody for 1 hr. Immunoreactive proteins were visualized using an Alpha Innotech Imaging Station (Cell Biosciences, Santa Clara, CA) and the Western Lightning ECL reagent (Perkin Elmer, Waltham, MA). Protein bands of interest were measured by densitometry using FluorChem 8800 software (Cell Biosciences, Santa Clara, CA). Membranes were stripped using Restore Western Blot Stripping Buffer (Thermo Scientific) and tested for removal of antibodies before re-probing with β-actin or α-tubulin. Changes in protein expression, normalized to β-actin or α-tubulin, were calculated as the mean difference in percentage compared to time-matched vehicle controls (0.1% DMSO), which were assigned a value of 100%.

#### TUNEL analysis in vitro

The terminal deoxynucleotidyl transferase dUTP nick end labeling method (TUNEL) was applied to CEM treated with 0–15 µM DIM for 48 hr. The *In Situ* Cell Death Detection kit with Fluorescein (Roche Applied Science, Indianapolis, IN) was used to label DNA strand breaks and the Guava Express Plus program was used to sort and quantify the amount of TdT incorporation. Detailed methods, including sample preparation and fluorescent microscopy, are provided in File S1. The apoptotic index (AI) was calculated from the flow cytometry results as follows: AI = (number of TUNEL-positive cells/total number of cells)×100.

#### Quantitative PCR for apoptosis pathway

Total RNA was extracted using TRIZOL reagent (Sigma-Aldrich) as recommended by the manufacturer from triplicate samples of CEM cells treated with 7.5 µM DIM for 4 or 24 hr. cDNA synthesis was performed using 2 µg RNA per sample with the RT^2^ First Strand Synthesis Kit (SABiosciences, Frederick, MD); quantitative PCR analysis for 84 genes related to human apoptosis was performed using the RT^2^ Profiler PCR Array System (SABiosciences) with the iCYCLER iQ5 Real-Time PCR System (Bio-Rad, Hercules, CA). Relative gene expression was calculated using the ΔΔC_t_ method [28] with the housekeeping genes *B2M* and *GAPDH* selected for normalization. Transcripts were considered absent if C_t_>35 and were removed from analysis.

### *In vivo* xenograft study with human CEM cells

#### Animal care and diet preparation

All protocols for the handling and treatment of mice were reviewed and approved by the Oregon State University Institutional Animal Care and Use Committee (Animal Care and Use Protocol #3837). Male NOD.CB17-*Prkdc^scid^*/SzJ (SCID) mice were purchased from Jackson Laboratories (Bar Harbor, ME) at 7 weeks of age and housed at the Laboratory Animal Resource Center at Oregon State University under controlled conditions of 20±1°C and 50±10% humidity with a 12∶12 hr light/dark cycle in micro-isolator cages (Super Mouse750™ Micro-Isolator ™, Life Products, Seaford, DE) with CareFRESH bedding. Mice were acclimated for one-week prior to any experimental procedures. Experimental diets were prepared by incorporating 500 or 2000 mg I3C, or 350 mg BioResponse-DIM (contains 100 mg DIM) per kg of powdered AIN93G diet (Research Diets, New Brunswick, NJ). All prepared diets were γ-irradiated (2.5 mRads) and stored at −20°C, protected from light throughout the course of the study.

#### CEM cell xenograft study

Detailed methods for the xenograft study are provided in File S1. Briefly, CEM cells were freshly collected, prepared in a 1∶1 (v/v) solution of medium/Matrigel, and engrafted subcutaneously (10^7^ cells/site) into SCID mice. Mice were fed diets containing 500 ppm I3C, 2000 ppm I3C, or 100 ppm DIM (350 ppm BioResponse-DIM) *ad libitum* for one-week prior to engraftment and throughout the course of the study. Xenograft measurements were conducted every third day with digital calipers, and tumor volume was estimated using the equation for an ellipsoid (L×W^2^×π/6).

#### TUNEL analysis of human CEM cell xenografts

Detailed methods for staining and analysis of xenograft tissues by TUNEL for detection of apoptosis are provided in File S1. Briefly, serial sections of xenografts were stained using the *In Situ* Cell Death Detection kit, POD (Roche Applied Science) with few modifications from the manufacturer's protocol. The apoptotic-index (AI) was calculated as follows: AI = (manual count TUNEL positive/auto count negative)×100.

### Statistical analyses

GraphPad Prism 5 software (La Jolla, CA) was used for all statistical analyses. One or two-way ANOVA were performed as appropriate for the number of experimental factors being examined. Statistical significance was inferred when *p*<0.05 and was denoted in each figure as follows: *, *p*<0.05; **, *p*<0.01, and ***, *p*<0.001. Non-linear regression analyses were performed using the equation for exponential growth to determine the impact of experimental diet on the doubling time (DT) of CEM xenografts. DT was calculated as follows: DT = [(T_o_−T_i_)ln2]/ln(V_o_/V_i_) where *T*_i_ and *T_o_* represent the initial and final time points and *V_i_* and *V_o_* represent initial and final tumor volumes. A significant effect of DIM on gene expression was inferred when the relative fold change was greater than 1.5-fold (log_2_ R<−0.58 or >0.58) with a *p*-value<0.05 (Student's *t*-test) compared to time-matched controls.

## Results

### Impact of DIM treatment CEM cells *in vitro*

#### DIM and I3C inhibit proliferation of CEM cells

**Figure 1:**
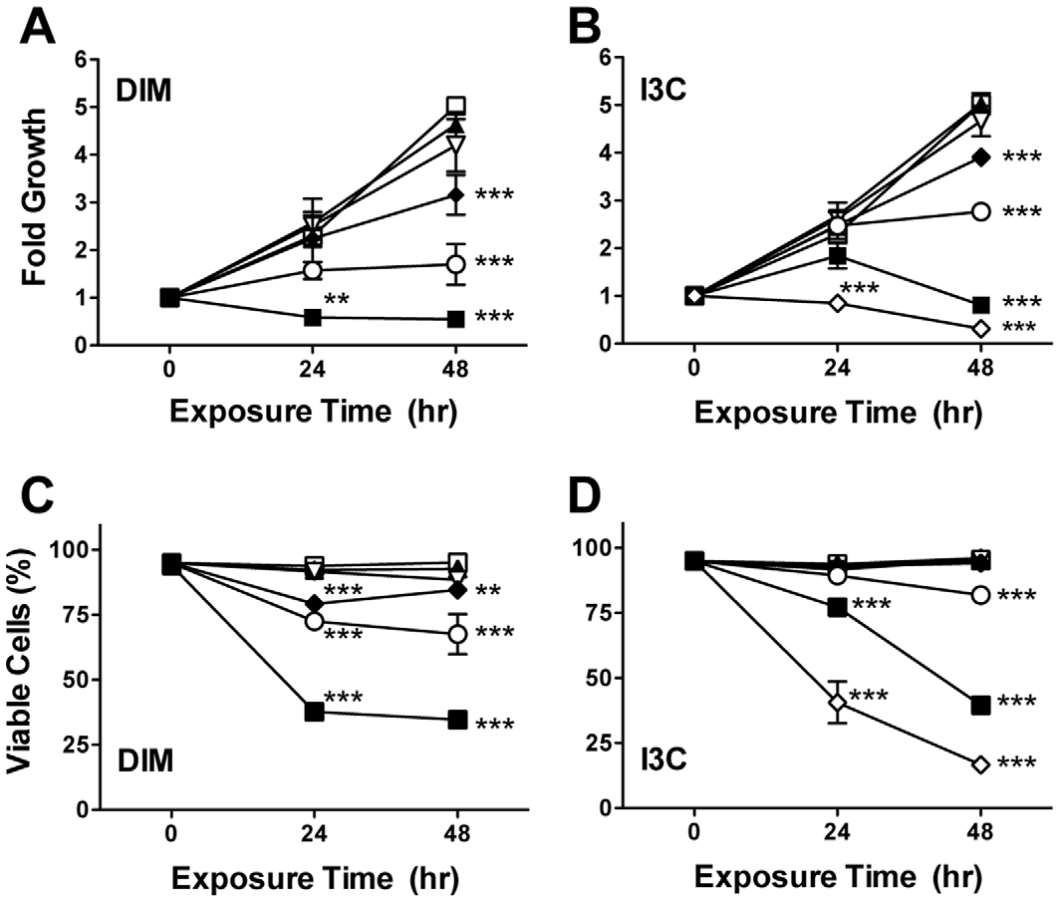
I3C and DIM reduce proliferation and viability of CEM cells. Cells were treated with 0 (□), 1.9 (▴), 3.8 (▿), 7.5 (⧫), 15 (○), or 30 (▪) µM DIM (panels A,C) or 0 (□), 15.6 (▴), 31.3 (▿), 62.5 (⧫), 125 (○), 250 (▪), or 500 (⋄) µM I3C (panels B,D) for 24 or 48 hr, then stained with ViaCount reagent for analysis of viable cell concentration and percent viability. Values are the mean fold change in cell proliferation (panels A, B) or percent viability (panels C, D) ± SEM (n = 3 independent experiments) normalized to control cells at 0 hr. **, *p*<0.01 and ***, *p*<0.001, as determined by two-way ANOVA with Bonferroni post-hoc test comparisons for significant effects of DIM treatments at each time point compared to time-matched vehicle control (0.1% DMSO).

To determine the impact of DIM and I3C on growth of a representative human T-ALL cell line, a time-course study was performed over a range of concentrations using CEM cells. DIM and I3C blocked the proliferation of CEM cells in a time- and concentration-dependent manner (Fig. 1A–B). Treatment with the highest concentrations of DIM or I3C significantly reduced CEM cell viability by up to 58 or 82%, respectively (Fig. 1C–D). Significant inhibition of proliferation and a decrease in viability was observed after 24 hr treatment with DIM or I3C, with the greatest response observed by 48 hr. However, I3C was substantially less effective, as much greater concentrations (>62.5 µM) were required to significantly reduce CEM cell growth or decrease viability compared to DIM (>7.5 µM).

#### Comparison of efficacy of DIM and I3C in multiple T-ALL cell lines

**Figure 2:**
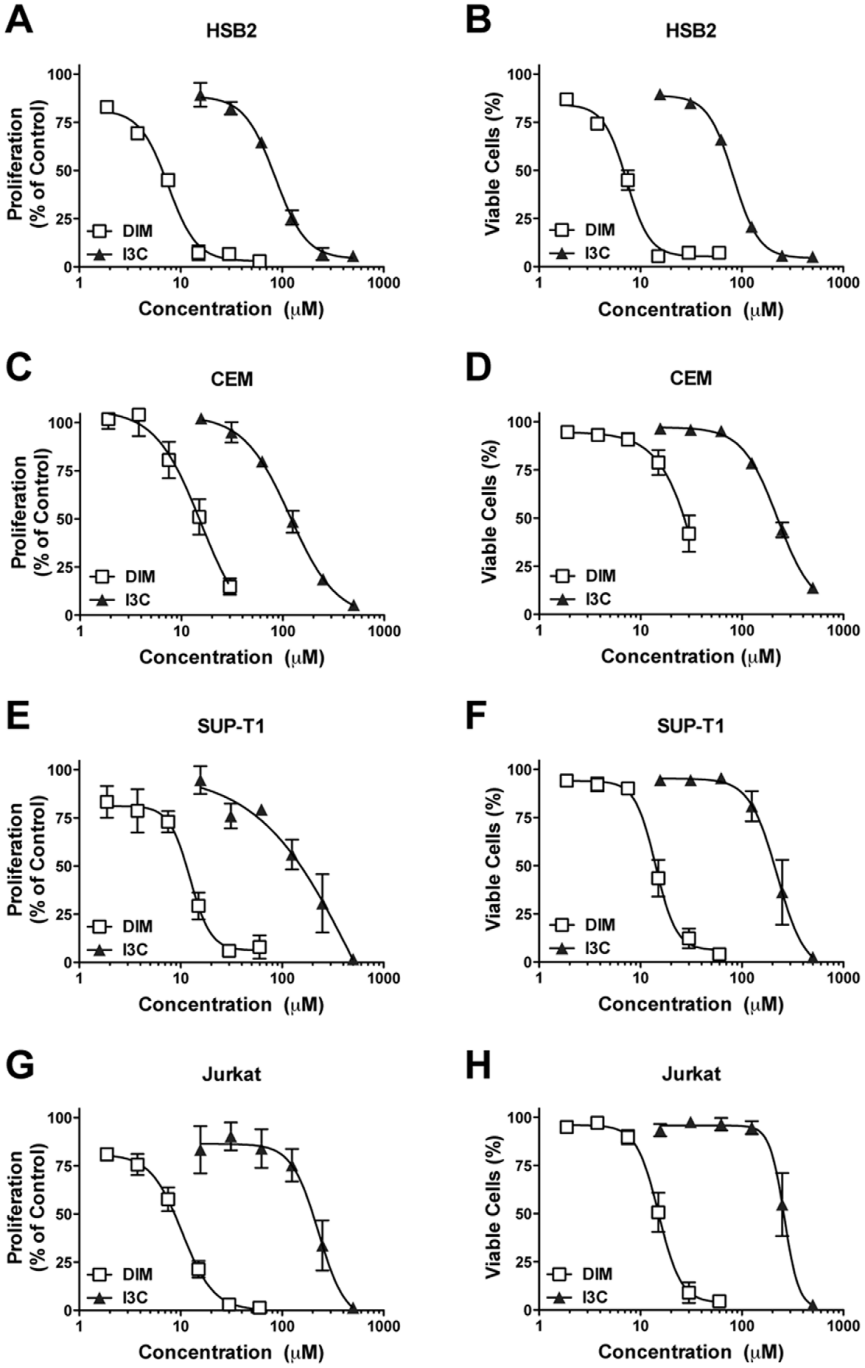
Comparison of I3C and DIM in multiple human T-ALL cell lines. Human CEM, HSB2, SUP-T1 and Jurkat cells were treated for 48 hr with I3C (15.6 up to 500 µM) or DIM (1.9 up to 60 µM), then stained with ViaCount reagent for analysis of cell concentration and percent viability. Values are the mean level of cell proliferation (panels A–D) or the mean percent viable cells (panels E–F) ± SEM (n = 3 independent experiments), normalized to the time-matched vehicle control (0.1% DMSO). Non-linear regression analysis (four parameter, variable slope) was performed (GraphPad Prism) to generate the concentration-response curve for each chemical in each cell line, from which IC_50_ values were obtained (see Table 2).

Concentration-response experiments were performed in four different T-ALL cell lines to determine whether DIM and I3C are similarly effective in reducing growth of T-ALL cells derived from T-cells at different stages of differentiation. In all cell types, *in vitro* treatment with DIM for 48 hr markedly reduced cell proliferation (IC_50_ values of 8 to 15 µM) and cell viability (IC_50_ values of 7 to 27 µM), whereas I3C was much less effective (proliferation IC_50_ values of 86 to 262 µM; viability IC_50_ values of 83 to 284 µM) (Fig. 2; Table 2). HSB2 cells were the most sensitive to inhibition of cell growth by DIM and I3C.

**Table 2:** Inhibition of T-ALL cell growth by DIM and I3C.

	IC_50_ (µM) for DIM		IC_50_ (µM) for I3C	
Cell line	Proliferation	Viability	Proliferation	Viability
CEM	15	27	122	223
HSB2	8	7	86	83
SUP-T1	13	14	262	284
Jurkat	9	15	228	222

*Note*: Non-linear regression analyses (four parameters, variable slope) were performed using data generated from each DIM and I3C concentration-response curve generated for each of the four cell lines tested (GraphPad Prism v5.0, San Diego, CA). IC_50_ values are the concentrations of DIM or I3C required to inhibit cell proliferation or viability by 50% compared to the vehicle control (0.1% DMSO).

#### DIM induces cell cycle arrest in CEM and HSB2 cells

**Figure 3:**
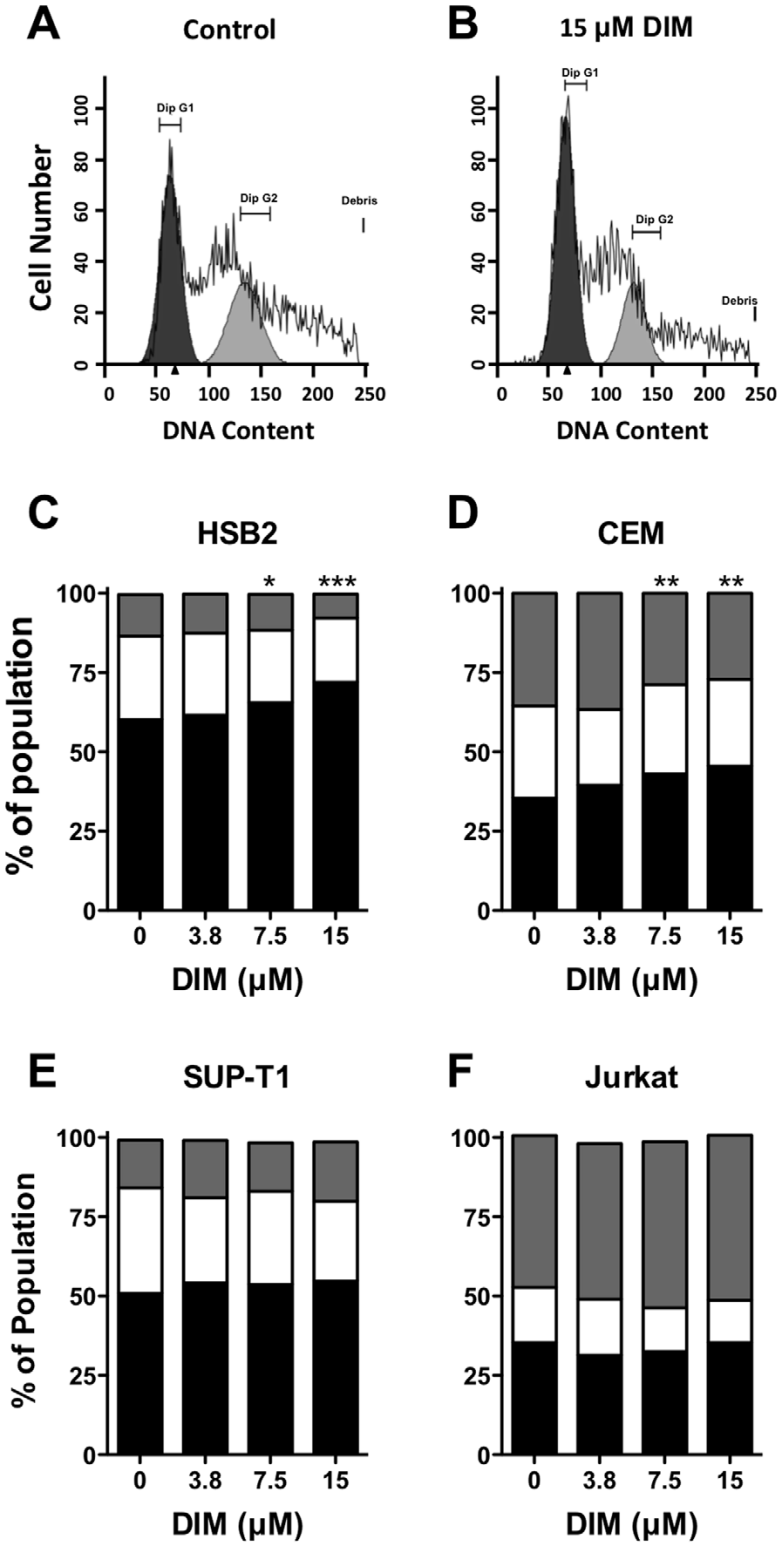
DIM induces cell-cycle arrest in CEM and HSB2 cells. Cells were treated with 0, 3.8, 7.5, or 15 µM DIM for 48 hr, then fixed in ice-cold 70% EtOH and stained with propidium iodide. DNA content distribution was analyzed by Guava PCA or Accuri C6 flow cytometry. (A–B) Representative histograms are shown for control and 15 µM DIM treatments at 48 hr in human CEM cells. (C–D) Distributions of CEM, HSB2, SUP-T1 or Jurkat cells in G_1_ (black), S (white), and G_2_ (grey) phases of cell-cycle progression at 48 hr (n = 3 to 5 independent experiments). *, *p*<0.05; **, *p*<0.01 or *** *p*<0.001 for G_1_ arrest compared to the vehicle control (0.1% DMSO) as determined by one-way RM ANOVA (matching by experiment day) with Dunnett's multiple comparisons post-hoc test.

The marked suppression of proliferation by DIM prompted us to evaluate cellular DNA content by flow cytometry in each of the four T-ALL cell lines. Treatment of CEM or HSB2 cells with 7.5 or 15 µM DIM for 48 hr resulted in a significant G_1_ cell cycle arrest, with substantially fewer cells progressing to the G_2_/M phase (Fig. 3). Shorter duration DIM treatment (6 and 12 hr) in CEM cells also caused a significant G_1_ arrest (data not shown). On the other hand, DIM treatment did not significantly alter cell cycle progression in either SUP-T1 or Jurkat cells (Fig. 3). Additionally, at the higher concentrations of DIM tested, a sub-G_1_ peak was observed in the raw histogram data (for example in CEM cells approximately 10% at 7.5 µM, and about 14% at 15 µM; data not shown) indicating an increasing population of apoptotic cells.

**Figure 4:**
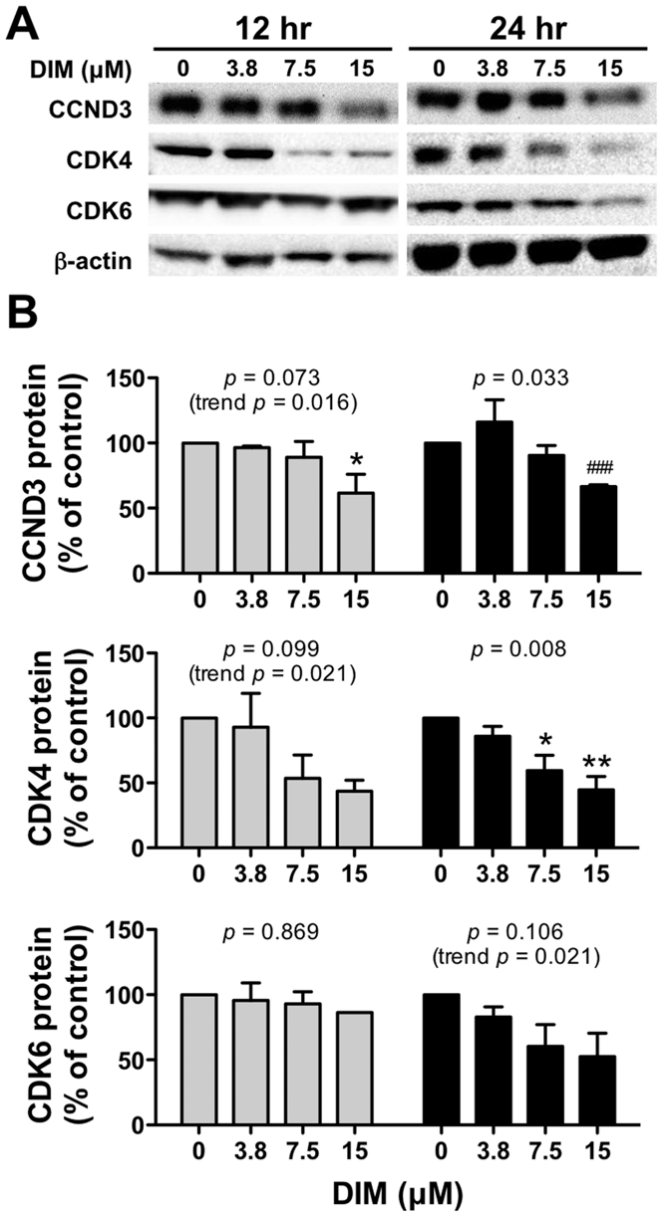
DIM reduces expression of cell-cycle regulatory proteins. Following either 12 hr (gray bars) or 24 hr (black bars) treatment with increasing concentrations of DIM, CEM cells were harvested and protein immunoassays were performed for detection of CCND3, CDK4 and CDK6 proteins (three replicate experiments performed). (A) A representative immunoblot is shown for each protein assay. (B) Values shown are average protein expression ± SEM normalized to β-actin, expressed as a percentage difference from time-matched vehicle controls (0.1% DMSO), which were assigned a value of 100%. *, *p*<0.05 or **, *p*<0.01 compared to 0 µM DIM (vehicle control) as determined by one-way ANOVA with Dunnett's post-hoc test for multiple comparisons; overall ANOVA *p*-values within each time group are indicated in each panel. In some cases where the *p*-value for the ANOVA was not <0.05, a significant linear trend was evident, as indicated by trend *p*-values in the figure. Finally, a Student's *t-*test (###, *p*<0.001) was performed to compare 15 µM DIM to vehicle control for CCND3 expression at 24 hr because high variability observed at the 3.8 µM concentration confounded the ANOVA post-hoc results (overall effect of DIM was significant).

Next, we measured the expression of key regulatory proteins of cell cycle progression by immunoassay in CEM cells. DIM suppressed expression of key cell cycle regulatory proteins *in vitro*, a finding that is consistent with DIM-induced G_1_ growth arrest (Fig. 4). Treatment with DIM for 12 or 24 hr decreased expression of CCND3 and CDK4 proteins in a concentration-dependent manner (*i.e.*, 38% and 56% decrease after treatment with 15 µM DIM for 24 hr, respectively), whereas a trend for decreasing CDK6 expression was evident (up to 48% decrease after 15 µM DIM for 24 hr). I3C also decreased expression of CCND3, CDK6 and CDK4 at 24 hr (data not shown), albeit at supra-physiological concentrations (>100 µM) that have been shown to be cytotoxic in healthy peripheral blood mononuclear cells [29].

#### DIM induces apoptosis in T-ALL cells

**Figure 5:**
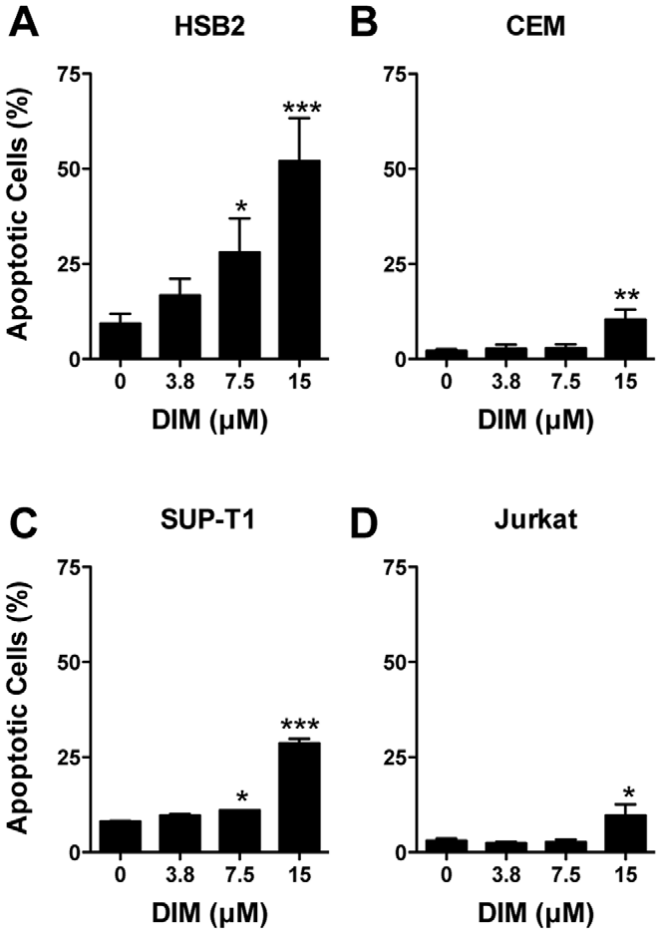
DIM induces apoptosis in human T-ALL cells. CEM, HSB2, SUP-T1 and Jurkat cells were treated with 3.8 to 15 µM DIM for 48 hr. Values are the proportion of apoptotic cells as determined using the ViaCount assay + SEM (n = 3 to 4 independent experiments). *, *p*<0.05; **, *p*<0.01 or *** *p*<0.001 for compared to the vehicle control (0 µM DIM, 0.1% DMSO) as determined by one-way RM ANOVA (matching by experiment day) with Dunnett's multiple comparisons post-hoc test.

Two methods for assessing the impact of DIM on apoptosis were used in this study. First, the portion of apoptotic cells following treatment with DIM for 48 hr was determined by the ViaCount assay. In all four T-ALL cell types, treatment with 15 µM DIM caused a significant increase in the percentage of apoptotic cells (Fig. 5), although the sensitivity to DIM varied with cell type. For example, HSB2 cells were the most sensitive to DIM-induced apoptosis (significant increase in apoptosis at DIM concentrations >7.5 µM up to 52%), whereas apoptosis was only modestly increased in Jurkat cells (10% apoptosis at 15 µM DIM).

**Figure 6:**
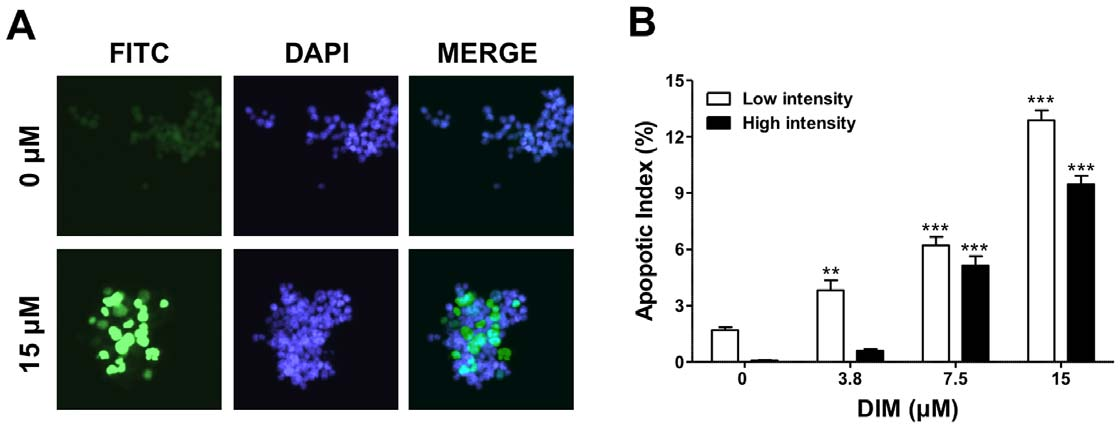
DIM induces apoptosis in CEM cells as detected by TUNEL. The *In situ* cell death detection kit (TUNEL) was applied to fixed CEM cells treated with 0 to 15 µM DIM for 48 hr. (A) Fluorescence images of control (0 µM) and DIM-treated (15 µM) cells were taken at 20× magnification following TUNEL labeling in mounting medium with DAPI. (B) Flow cytometry was used to identify and quantify cells with no, low (open bar) or high (solid bar) intensity staining. **, *p*<0.01 or ***, *p*<0.001 as determined by one-way ANOVA with Dunnett's post-hoc test comparisons for significant effects of DIM treatments within each intensity category as compared to vehicle control (0 µM DIM, 0.1% DMSO).

Next, the extent of DNA strand breaks *in vitro* was analyzed using the TUNEL method and a commercially available kit (*In Situ* Cell Death Detection Kit, Roche Applied Science) in the CEM cell line only. Marked incorporation of fluorescein-dUTP was evident by fluorescence microscopy (Fig. 6A). Fixed and stained samples were applied to a benchtop flow cytometer (Guava PCA) for quantitative analysis of results. This approach identified populations of both low and high TdT incorporation by fluorescence intensity, reflective of low and high cellular levels of apoptosis (Fig. 6B). For all concentrations of DIM tested, the low intensity apoptotic index increased in a concentration-dependent manner relative to controls, with the percentage of CEM cells undergoing low levels of apoptosis ranging from 2 to 13%. At higher concentrations (≥7.5 µM) of DIM, an increase in the number of cells with a high level of apoptotic response was also observed. Apoptosis was detected in 22% of the cell population at the highest concentration of DIM tested.

#### DIM alters expression of genes that regulate the apoptosis pathway

We determined the effects of DIM on expression of gene targets relevant for regulation of apoptosis in human cells. Fold change values and results of the statistical analyses for all gene targets on the apoptosis PCR pathway array are provided in Table S1. *In vitro* exposure to 7.5 µM DIM for 4 hr significantly altered the expression level of eight genes more than 1.5-fold (p<0.05) with respect to the time-matched controls (Table 3). This set of genes accounted for 9.5% of transcripts queried by the quantitative PCR apoptosis pathway array. Among those transcripts, BCL2L10, CD40LG, HRK, TNFRSR1A and TNFSF8 were significantly induced, while only TNF was repressed. Following 24 hr of DIM exposure, expression levels of CD40LG and HRK remained elevated, while TNFRSF25 and TRAF4 were significantly repressed (<−1.5-fold, *p*<0.05) (Table 3).

**Table 3:** DIM-induced changes in expression of select apoptosis-related genes in CEM cells. *

			Log_2_ R (*p*-value)†	
Unigene	Symbol	Description	4 hours	24 hours
Hs.283672	BCL2L10	BCL2-like 10 (apoptosis facilitator)	**1.05** (0.459)	**0.63** (0.023)
Hs.592244	CD40LG	CD40 ligand	**0.75** (0.019)	**0.62** (0.043)
Hs.87247	HRK	Harakiri, BCL2 interacting protein (contains only BH3 domain)	**0.88** (0.014)	**1.35** (0.004)
Hs.241570	TNF	Tumor necrosis factor (TNF superfamily, member 2)	**−0.63** (0.016)	−0.39 (0.026)
Hs.279594	TNFRSF1A	Tumor necrosis factor receptor superfamily, member 1A	**1.22** (0.002)	−0.46 (0.331)
Hs.462529	TNFRSF25	Tumor necrosis factor receptor superfamily, member 25	nd‡	**−1.15** (0.015)
Hs.654445	TNFSF8	Tumor necrosis factor (ligand) superfamily, member 8	**0.90** (0.026)	0.34 (0.009)
Hs.8375	TRAF4	TNF receptor-associated factor 4	nd	**−0.59** (0.017)

* A complete list of DIM-induced changes in gene expression, including all genes on the RT^2^ Profiler Apoptosis array, is provided in Table S1.

† Log_2_ fold change (R) values are highlighted in bold if level of change is >1.5-fold (Log_2_ R<−0.58 or >0.58) compared to vehicle (0.1% DMSO) control. *p*-values were determined by a Student's *t*-test assuming equal variances.

‡ nd, not detected by RT^2^ PCR profiler array at this time point (C_t_>35).

### Impact of DIM and I3C treatment on growth of CEM xenografts

#### DIM and I3C inhibit growth of CEM xenografts in vivo

**Figure 7:**
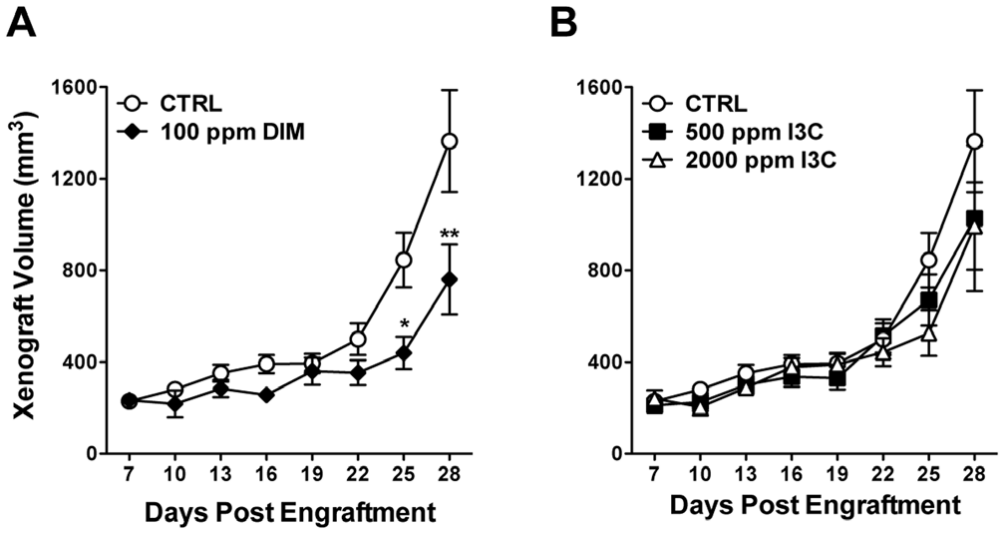
DIM and I3C suppress CEM cell xenograft growth. Male NOD.CB17-*Prkdc^scid^*/SzJ mice were engrafted with CEM cells as described in *Materials and Methods* and fed control diet (CTRL), 100 ppm DIM (panel A) or 500 or 2000 ppm I3C (panel B) for 28 days. Growth of xenografts was assessed every third day and compared to nodule volumes in control-fed animals. *, *p*<0.05 or **, *p*<0.01 as determined by two-way repeated measures ANOVA with Bonferroni post-hoc tests to evaluate the effects of diet on tumor growth at each time point compared to the time-matched control. *p*-values for overall effect of each treatment on tumor growth compared to control are: 100 ppm DIM, *p* = 0.041; 500 ppm I3C, *p* = 0.356; and 2000 ppm I3C, *p* = 0.271.

Another key objective of this study was to determine whether dietary DIM or I3C reduced the growth of human CEM cells *in vivo* using a SCID mouse xenograft model. The rate of body weight gain or average final body weight was not significantly affected by any of the dietary treatments (Fig. S1). On average, animals in the DIM group consumed about 0.4 mg DIM/day, based on per cage diet consumption data (Fig. S1B). The rate of successful CEM engraftment in this study was high (57/59 animals), with solid nodules palpable within one week (approximately 250 mm^3^). In this study, short-term (1 week pre-engraftment+28 days post-engraftment; 35 days total) dietary treatment with 100 ppm DIM, 500 ppm I3C or 2000 ppm I3C did not significantly affect average body weight or rate of body weight gain (Fig. S1A). Animals fed 500 ppm I3C apparently consumed less food on a daily basis compared to the other diet groups (Fig. S1B.); one animal was removed from this group due to an unrelated health problem. Tumor volume in control-fed animals increased by about 600%, with an average doubling time (DT) of 6.4 days (Fig. 7; Table 4). Dietary DIM significantly reduced growth of CEM xenografts (*p* = 0.041, two-way RM ANOVA), and a significant effect of dietary DIM on CEM nodule size was detected by day 25 (*p*<0.05, Bonferroni's post-hoc tests compared to control) (Fig. 7A). At the conclusion of the study, the final average tumor size in DIM-treated animals was substantially and significantly reduced (44% decrease in volume) compared to control animals. Moreover, the rate of growth of CEM cell xenografts in animals fed 100 ppm DIM was significantly slower with a DT of 10.2 days (Table 4) compared to 6.4 days for control fed animals (*p*<0.001 by one-way ANOVA). I3C was less effective at reducing xenograft growth; 500 and 2000 ppm diet concentrations decreased tumor volume by 25% or 27% by day 28, respectively, although these levels of effect were not statistically significant (500 ppm I3C, *p* = 0.356; 2000 ppm I3C, *p* = 0.271, by two-way RM ANOVA) (Fig. 7B). However, tumor growth rate was significantly reduced by 2000 ppm I3C, with a calculated doubling time of 8.5 days (*p* = 0.006) (Table 4).

**Table 4:** Growth of human CEM cell xenografts in SCID mice fed DIM or I3C.

Treatment	Final tumor volumemm^3^ ± SEM	Tumor doubling time†Days (95% CI)
Control	1360±222	6.43 (5.22–8.35)
100 ppm DIM (350 ppm BR-DIM)	761±153 **	10.2 (7.51–15.9) ###
500 ppm I3C	1030±318	7.57 (5.43–12.5)
2000 ppm I3C	994±191	8.45 (6.47–12.2) ##

*Note:*

** , *p*<0.01 as determined by two-way ANOVA with Dunnett's post-hoc test comparisons for significant effect of experimental diet compared to the time-matched control (day 28 values for tumor volume are shown).

## , *p*<0.01 or

### , *p*<0.001 as determined by one-way ANOVA with Dunnett's post-hoc test comparisons for significant effects of experimental diets compared to control.

† Tumor growth rates were modeled by non-linear regression analyses using the exponential growth equation with least-squares fit (Prism 5). Average doubling time (DT) values are shown and were calculated as follows: DT = [(T_o_−T_i_)×ln2]/ln(V_o_/V_i_) where *T*_i_ and *T_o_* represent the initial and final time points and *V_i_* and *V_o_* represent initial and final tumor volumes. *p*-values (extra sum of squares F test) are reported for comparison of calculated growth curves for indicated treatments compared to control diet.

#### Dietary DIM induces apoptosis in CEM xenografts

**Figure 8:**
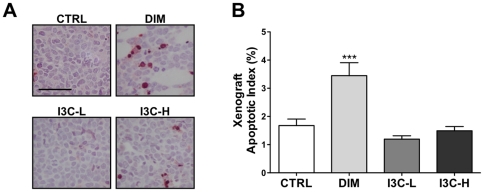
DIM induces apoptosis *in vivo*. (A) The *In situ* cell death detection kit (TUNEL) was applied to xenograft sections following exposure to control diet (CTRL), 100 ppm DIM, 500 ppm I3C (I3C-L), or 2000 ppm I3C (I3C-H). Dark staining indicates apoptotic cells, and the scale bar represents 50 µm. (B) Manual and software-assisted counting was performed for xenograft sections as described in File S1 to calculate the percentage of positive cells. ***, *p*<0.001 as determined by one-way ANOVA with Dunnett's multiple comparisons post-hoc test.

Because high rates of apoptosis were detected in CEM cells exposed to DIM *in vitro*, apoptosis was also assessed in CEM xenografts following dietary exposure to 100 ppm DIM, 500 ppm I3C, and 2000 ppm I3C (Fig. 8A). Dietary DIM resulted in a significant (*p*<0.001), two-fold increase in the number of TUNEL-positive cells in mice fed DIM (3.4±0.5%) compared to control mice (1.7±0.2%). Alternatively, AI values for xenograft sections from mice exposed to 500 or 2000 ppm I3C were not significantly different from control (Fig. 8B).

## Discussion

We provide evidence for the first time that DIM significantly impairs the growth of human T-ALL cells *in vitro* and *in vivo*. Moreover, we show that DIM blocks growth of T-ALL cell types that represent the spectrum of T-cell differentiation arrest occurring within this disease, ranging from least differentiated to nearly mature (HSB2>CEM>SUP-T1>Jurkat). All four T-ALL cell types studied responded to DIM treatment in a dose-dependent manner, as shown by inhibition of cell proliferation and viability and increased levels of apoptosis; in addition, a G_1_ cell cycle arrest was observed in HSB2 and CEM cells, lines that represent early (pre-T) differentiated cells. In this study, we also show that the I3C derivative, DIM, was far more potent than its precursor and exhibited therapeutic effects on a variety of highly aggressive juvenile T-ALL cell lines, including Jurkat and CEM, at physiological concentrations. Others have reported that I3C suppressed NFκB stimulation by TNF and downstream gene products, including CCND1, BCL-2 and TRAF1, in myeloid and leukemia (Jurkat) cells [30], but I3C was not capable of blocking the growth of T-cell lines that were not infected with human T-cell leukemia virus type-1 (MOLT-4, Jurkat and CCRF-CEM) [29].

The SCID mouse model supports the solid growth of subcutaneously injected human acute leukemia blast cells in a manner that is easily measurable and exhibits a dissemination pattern analogous to the human disease [31], [32]. We supplemented this pre-clinical model with dietary indoles to determine the extent of xenograft growth suppression following absorption, metabolism and disposition to the grafted cells. This study is the first to employ continuous exposure of I3C or DIM through the diet, as opposed to bolus administration via gavage or injection, with a human cell xenograft model in SCID mice. In the present study, growth of human CEM cell xenografts in mice consuming DIM (approximately 0.4 mg/day) was only about half that of the control animals, an observation that is comparable to other studies with breast cancer cell xenografts that employed even greater amounts of DIM or more direct routes of exposure. For example, oral gavage of about 1 mg DIM/day (3.5 mg BioResponse-DIM/day) decreased growth of MDA-MB-231 xenografted cells by approximately 30% after 3 weeks of exposure [33] whereas daily 5 mg/kg *s.c.* injections of DIM at the site of MCF-7 xenografts reduced tumor volume by about 45% [34].

We selected a dietary concentration of 2000 ppm I3C level based on the apparent anticancer effects at this level observed in our previous studies [26], [35]. The likely proportion of this I3C diet to be converted to DIM following *in vivo* condensation corresponds to a diet concentration of 350 ppm DIM, based on a 2∶1 molar ratio and assuming a 20% conversion rate [20], [23]. The BioResponse-DIM formulation is a commercially available dietary supplement, sold for human consumption, that is also used in animal studies and clinical trials; thus, 350 ppm BioResponse-DIM (100 ppm DIM) was selected in anticipation of comparable bioactivity to the 2000 ppm I3C treatment [23]. Pharmacokinetic studies in mice comparing this formulated DIM to crystalline DIM demonstrate a 50% improvement in adsorption [23]. This difference in bioavailability, along with the rapid elimination of I3C and formation of additional bioactive I3C derivatives, may account for the reduced efficacy of 2000 ppm I3C *in vivo* compared to 100 ppm DIM.

DIM was also significantly more potent than I3C *in vitro* based on the relative IC_50_ values for inhibition of cell proliferation and viability across all cell lines tested. Moreover, the anti-proliferative effect of I3C was delayed compared to DIM, suggesting that conversion of I3C to DIM and other ACPs in the culture media may contribute to the physiological effects of I3C. A recent report by Bradlow and Zeligs [36] showed that addition of 100 µM I3C to culture media at a neutral pH resulted in concentrations of DIM of about 25 µM within 24 hours. However, because the degree of difference in potency of DIM and I3C varied across the four T-ALL cell lines tested and by the endpoint examined (viability, proliferation) in this study, the apparent lower potency of I3C compared to DIM cannot be fully explained by conversion of I3C to DIM in the culture media.

Other plausible explanations exist for the distinctive responses to DIM observed in the four T-ALL cell lines studied, which are characterized by different lineages of T-cell differentiation (pre-T, cortical-T and mature-T), as well as different ages and genders of the source patients (Table 1). Gene deletions and mutations as well as epigenetic mechanisms of gene dysregulation are commonly implicated in the oncogenesis of T-cells and in therapeutic outcome [37], [38], [39]. Common leukemic signature genes include those involved in normal T-cell receptor signaling and T-cell differentiation such as *NOTCH1*, *NOTCH3*, *HOX11*, *TAL1*, *LYL1* and *LMO1* [5], [37]. A selection of these therapeutically relevant targets and their status in HSB2, CEM, SUP-T1 and Jurkat cells are listed in Table 1.

Although beyond the scope of this study, the different combinations of these aberrations across the cell lines tested are likely to play a role in the therapeutic effect of DIM. The variable responses to both targeted and conventional chemotherapeutic drugs that have been previously observed in T-ALL cells are likely a consequence of the respective mutations harbored by the cell lines (e.g., [11], [40]). Thus, the observation that DIM had variable potency for blocking growth of T-ALL cells (though effective in all four cell lines tested) is not necessarily unexpected given the apparent variability in response of T-ALL cells to drug therapies.

Treatment of CEM and HSB cells with DIM caused a blockade of cell-cycle progression at the G_1_ phase checkpoint, although this effect was not observed in more differentiated T-ALL cell lines (SUP-T1 or Jurkat); DIM (and I3C) also suppressed expression of CCND3, CDK4 and CDK6 cell cycle regulatory proteins in CEM cells. Early progression of the eukaryotic cell cycle is positively regulated by the coupling of D-type cyclins with the highly homologous CDK4 or CDK6 proteins and negatively regulated by cyclin dependent kinase inhibitors and phosphatases [41]. I3C and DIM inhibit proliferation and cell cycle progression of various tumor cells, including breast [42], prostate [43] and colon [44], via down-regulation of cyclins and cyclin dependent kinases and/or up-regulation of cyclin dependent kinase inhibitors, such as p21 or p27 [42], [43], [45]. The *INK4A* gene locus, which encodes the cyclin dependent kinase inhibitors p16 and p19, is inactivated in up to 80% of T-ALL cases and in all T-ALL cell lines tested [46], [47]. CDK6 is the initial CDK induced during T-lymphocyte activation/proliferation and is highly expressed in T-cell lymphoblastic leukemias/lymphomas [48]; similarly, over-expression of cyclin D3 is oncogenic in an array of mouse and human T-ALL cell lines [49]. Aberrant expression of cyclin D3 and cyclin dependent kinases during leukemic transformation underscores their relevance as therapeutic targets for I3C/DIM.

DIM treatment effectively induced apoptosis in human T-ALL cells *in vitro* and *in vivo*, although the apoptotic response to *in vitro* treatment with DIM varied greatly among the cell lines tested. HSB2 cells, which represent T-ALL originating from T-cells at a very early stage of differentiation, were highly sensitive to DIM-induced apoptosis compared to other T-ALL cell types that were only modestly affected. This observation reinforces a general conclusion of this report that HSB2 cells are more sensitive to the anticancer effects of DIM *in vitro*. DIM treatment of CEM cells *in vitro* also altered expression of mRNA transcripts belonging to the BCL-2 superfamily or involved in TNF signaling, suggesting the involvement of both intrinsic and extrinsic apoptotic pathways. Others have shown that *in vitro* treatment with I3C or DIM inhibits NFκB activity in human breast and prostate cancer cells undergoing apoptosis [18], [50] and reduces BCL-2 mRNA and protein expression in breast cancer cells [51]. Moreover, expression of HRK (a BH3 domain-only BCL2 family member) is induced in hematopoietic progenitor cells upon growth factor removal or chemotherapeutic administration [52]. In this study, treatment of CEM cells with 7.5 µM DIM rapidly and continuously elevated HRK expression *in vitro*, which represents a putative and novel therapeutic target of these dietary indoles.

Bioactive dietary components may be utilized as part of a healthy lifestyle aimed at disease prevention or therapy. The ability of I3C/DIM to target multiple pro-survival pathways in cancer cells, while causing few adverse effects on normal cells, has been explored in a number of cancer models with substantial success. Collectively, our work points to the potential benefit of exposure to these agents at early life stages for chemoprotection, from gestation through adolescence, when leukemia is most prevalent [26, this study]. Based on available human and animal data [22], [23], the concentrations of DIM used in this study *in vitro* are likely achievable *in vivo*. In conclusion, our observations suggest that DIM may be a beneficial chemotherapeutic agent or adjunct therapy for T-ALL patients.

## Supporting Information

Figure S1 **Body weight gain and food consumption of mice engrafted with human CEM cells and fed DIM or I3C diets.** Male NOD.CB17-*Prkdc^scid^*/SzJ mice were fed diets containing 100 ppm DIM (350 BR-DIM, ⧫), 500 ppm I3C (▵, I3C-L), 2000 ppm I3C (○, I3C-H) or control diet (▪) throughout the xenograft study. (A) Following engraftment with CEM cells, animals were weighed every third day to monitor the rate of weight gain. A significant effect of experimental diet was not observed on weight gain, as determined by a two-way repeated-measures ANOVA (source of variation and *p*-value: diet treatment, *p* = 0.543; time, *p*<0.0001; interaction, *p* = 0.053; subjects matching, *p*<0.001). (B) Average food intake was assessed daily on a per cage basis (two subjects per cage). *, *p*<0.05 as determined by one-way ANOVA with Dunnett's post-hoc multiple comparisons test compared to control (CTRL) diet.
(TIF)

Table S1 **DIM-induced changes in expression of genes associated with apoptosis pathway in human CEM cells.**
(PDF)

File S1 **Supplemental methods.** Additional details on experimental methods are provided, including TUNEL analysis of CEM cells cultured *in vitro*, the CEM cell xenograft study and TUNEL analysis of human CEM cell xenografts.
(PDF)
