# Supplementary figures and images for: 3,3′-Diindolylmethane Induces G1 Arrest and Apoptosis in Human Acute T-Cell Lymphoblastic Leukemia Cells
Source: PLoS One. 2012 Apr 13;7(4):e34975. doi: 10.1371/journal.pone.0034975 (PMC3325915; doi:10.1371/journal.pone.0034975)

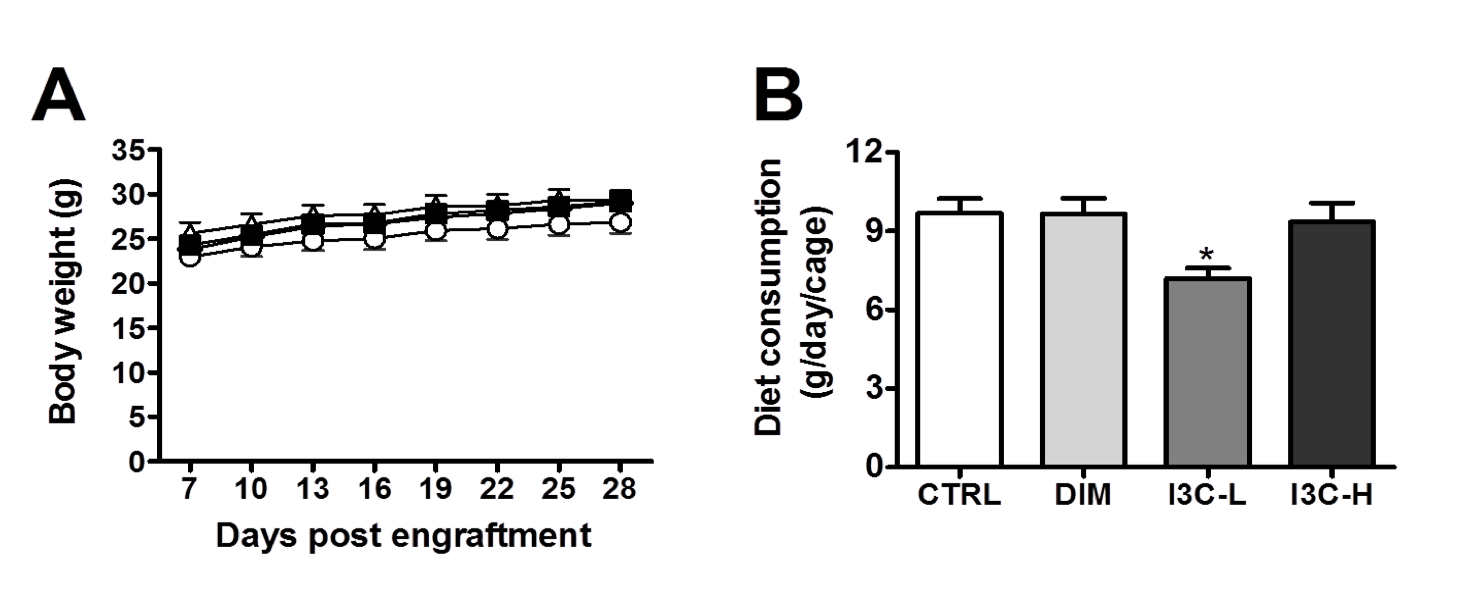

Supplement: Figure S1 — Body weight gain and food consumption of mice engrafted with human CEM cells and fed DIM or I3C diets. Male NOD.CB17-Prkdcscid/SzJ mice were fed diets containing 100 ppm DIM (350 BR-DIM, ⧫), 500 ppm I3C (▵, I3C-L), 2000 ppm I3C (○, I3C-H) or control diet (▪) throughout the xenograft study. (A) Following engraftment with CEM cells, animals were weighed every third day to monitor the rate of weight gain. A significant effect of experimental diet was not observed on weight gain, as determined by a two-way repeated-measures ANOVA (source of variation and p-value: diet treatment, p = 0.543; time, p<0.0001; interaction, p = 0.053; subjects matching, p<0.001). (B) Average food intake was assessed daily on a per cage basis (two subjects per cage). *, p<0.05 as determined by one-way ANOVA with Dunnett's post-hoc multiple comparisons test compared to control (CTRL) diet. (TIF) [file pone.0034975.s001.tif]
