# Supplementary material for: 3,3′-Diindolylmethane Induces G1 Arrest and Apoptosis in Human Acute T-Cell Lymphoblastic Leukemia Cells
Source: PLoS One. 2012 Apr 13;7(4):e34975. doi: 10.1371/journal.pone.0034975 (PMC3325915; doi:10.1371/journal.pone.0034975)
Supplement: Table S1 — DIM-induced changes in expression of genes associated with apoptosis pathway in human CEM cells. (PDF) [file pone.0034975.s002.pdf]

Table S1. DIM-induced changes in expression of genes associated with apoptosis pathway in human CEM cells

			Length of treatment †			
			4 hours		24 hours	
**Unigene**	**Symbol** *	**Description**	**Log_2 R**	***p*-value**	**Log_2 R**	***p*-value**
TNF ligand family						
Hs.592244	**CD40LG**	CD40 ligand	**0.75**	**0.019**	**0.62**	**0.043**
Hs.2007	FASLG	Fas ligand (TNF superfamily, member 6)	0.56	0.153	0.37	0.612
Hs.36	LTA	Lymphotoxin alpha (TNF superfamily, member 1)	nd ‡	nd	0.14	0.258
Hs.241570	**TNF**	Tumor necrosis factor (TNF superfamily, member 2)	**-0.63**	**0.016**	-0.39	0.026
Hs.478275	TNFSF10	Tumor necrosis factor (ligand) superfamily, member 10	0.38	0.123	0.23	0.421
Hs.501497	CD70	CD70 molecule	0.00	0.981	-0.04	0.852
Hs.654445	**TNFSF8**	Tumor necrosis factor (ligand) superfamily, member 8	**0.90**	**0.026**	0.34	0.009
TNF Receptor & Death Domain Family						
Hs.472860	CD40	CD40 molecule, TNF receptor superfamily member 5	**0.70**	0.521	**-1.12**	0.255
Hs.244139	FAS	Fas (TNF receptor superfamily, member 6)	0.21	0.160	0.00	0.993
Hs.1116	LTBR	Lymphotoxin beta receptor (TNFR superfamily, member 3)	0.25	0.811	**-0.70**	0.542
Hs.279594	**TNFRSF1A**	Tumor necrosis factor receptor superfamily, member 1A	**1.22**	**0.002**	-0.46	0.331
Hs.654459	TNFRSF9	Tumor necrosis factor receptor superfamily, member 9	**0.70**	0.475	**-1.22**	0.068
Hs.591834	TNFRSF10A	Tumor necrosis factor receptor superfamily, member 10a	-0.11	0.822	-0.11	0.866
Hs.521456	TNFRSF10B	Tumor necrosis factor receptor superfamily, member 10b	0.41	0.067	0.50	0.072
Hs.81791	TNFRSF11B	Tumor necrosis factor receptor superfamily, member 11b	**-1.02**	0.253	**-1.56**	0.136
Hs.443577	TNFRSF21	Tumor necrosis factor receptor superfamily, member 21	0.06	0.384	-0.31	0.149
Hs.462529	**TNFRSF25**	Tumor necrosis factor receptor superfamily, member 25	nd	nd	**-1.15**	**0.015**
Hs.355307	CD27	CD27 molecule	0.15	0.142	0.01	0.904
Hs.380277	DAPK1	Death-associated protein kinase 1	-0.08	0.671	-0.21	0.162
Hs.86131	FADD	Fas (TNFRSF6)-associated via death domain	0.12	0.603	-0.48	0.057
Hs.460996	TRADD	TNFRSF1A-associated via death domain	0.00	1.000	0.10	0.492
Bcl-2 family						
Hs.370254	BAD	BCL2-associated agonist of cell death	0.36	0.083	**-0.66**	0.283
Hs.377484	BAG1	BCL2-associated athanogene	-0.46	0.024	-0.25	0.084
Hs.523309	BAG3	BCL2-associated athanogene 3	0.07	0.559	0.01	0.907
Hs.194726	BAG4	BCL2-associated athanogene 4	0.03	0.830	-0.42	0.130
Hs.485139	BAK1	BCL2-antagonist/killer 1	0.11	0.615	-0.30	0.068
Hs.624291	BAX	BCL2-associated X protein	0.21	0.168	0.16	0.536
Hs.150749	BCL2	B-cell CLL/lymphoma 2	0.28	0.074	-0.15	0.433
Hs.227817	BCL2A1	BCL2-related protein A1	-0.19	0.379	0.32	0.539
Hs.516966	BCL2L1	BCL2-like 1	**0.61**	0.081	-0.31	0.606
Hs.283672	**BCL2L10**	BCL2-like 10 (apoptosis facilitator)	**1.05**	0.459	**0.63**	**0.023**
Hs.469658	BCL2L11	BCL2-like 11 (apoptosis facilitator)	-0.51	0.113	-0.04	0.799
Hs.410026	BCL2L2	BCL2-like 2	0.24	0.134	-0.01	0.945
Hs.486542	BCLAF1	BCL2-associated transcription factor 1	-0.36	0.016	-0.41	0.080
Hs.591054	BID	BH3 interacting domain death agonist	-0.31	0.073	-0.21	0.153

Table S1. DIM-induced changes in expression of genes associated with apoptosis pathway in human CEM cells

			Length of treatment [†]			
			4 hours		24 hours	
**Unigene**	**Symbol** [*]	**Description**	**Log_2 R**	***p*-value**	**Log_2 R**	***p*-value**
Hs.475055	BIK	BCL2-interacting killer (apoptosis-inducing)	-0.11	0.656	**-0.71**	0.066
Hs.145726	BNIP1	BCL2/adenovirus E1B 19kDa interacting protein 1	0.19	0.311	**-0.70**	0.058
Hs.646490	BNIP2	BCL2/adenovirus E1B 19kDa interacting protein 2	0.21	0.211	0.00	0.977
Hs.144873	BNIP3	BCL2/adenovirus E1B 19kDa interacting protein 3	-0.38	0.054	-0.58	0.004
Hs.131226	BNIP3L	BCL2/adenovirus E1B 19kDa interacting protein 3-like	-0.10	0.645	-0.18	0.081
Hs.87247	HRK	Harakiri, BCL2 interacting protein (contains only BH3 domain)	**0.88**	**0.014**	**1.35**	**0.004**
Hs.632486	MCL1	Myeloid cell leukemia sequence 1 (BCL2-related)	-0.38	0.077	-0.20	0.077
Caspase family & death effectors						
Hs.2490	CASP1	Caspase 1, apoptosis-related cysteine peptidase (interleukin 1, beta, convertase)	-0.07	0.576	0.25	0.117
Hs.5353	CASP10	Caspase 10, apoptosis-related cysteine peptidase	0.51	0.018	0.23	0.067
Hs.466057	CASP14	Caspase 14, apoptosis-related cysteine peptidase	**-0.82**	0.294	**-0.65**	0.529
Hs.368982	CASP2	Caspase 2, apoptosis-related cysteine peptidase	0.00	0.981	-0.48	0.000
Hs.141125	CASP3	Caspase 3, apoptosis-related cysteine peptidase	0.14	0.687	-0.33	0.010
Hs.138378	CASP4	Caspase 4, apoptosis-related cysteine peptidase	0.45	0.275	0.15	0.239
Hs.213327	CASP5	Caspase 5, apoptosis-related cysteine peptidase	**0.84**	0.556	**-0.96**	0.361
Hs.654616	CASP6	Caspase 6, apoptosis-related cysteine peptidase	-0.04	0.770	-0.04	0.673
Hs.9216	CASP7	Caspase 7, apoptosis-related cysteine peptidase	0.01	0.918	-0.23	0.083
Hs.599762	CASP8	Caspase 8, apoptosis-related cysteine peptidase	0.40	0.215	0.18	0.668
Hs.329502	CASP9	Caspase 9, apoptosis-related cysteine peptidase	0.23	0.060	0.30	0.409
Hs.390736	CFLAR	CASP8 and FADD-like apoptosis regulator	0.20	0.369	0.04	0.779
**IAP family**						
Hs.710305	NAIP	NLR family, apoptosis inhibitory protein	0.12	0.382	0.06	0.424
Hs.696238	BIRC2	Baculoviral IAP repeat-containing 2	0.41	0.029	0.16	0.168
Hs.127799	BIRC3	Baculoviral IAP repeat-containing 3	0.48	0.016	0.04	0.743
Hs.150107	BIRC6	Baculoviral IAP repeat-containing 6	-0.28	0.053	-0.14	0.292
Hs.348263	BIRC8	Baculoviral IAP repeat-containing 8	**-0.82**	0.294	**-1.01**	0.325
Hs.356076	XIAP	X-linked inhibitor of apoptosis	0.28	0.171	-0.16	0.210
**TRAF family**						
Hs.522506	TRAF2	TNF receptor-associated factor 2	**1.14**	0.166	**-1.90**	0.054
Hs.510528	TRAF3	TNF receptor-associated factor 3	**1.17**	0.011	**-1.42**	0.112
Hs.8375	TRAF4	TNF receptor-associated factor 4	nd	nd	**-1.51**	**0.017**
**CARD family**						
Hs.552567	APAF1	Apoptotic peptidase activating factor 1	0.25	0.140	-0.46	0.068
Hs.193516	BCL10	B-cell CLL/lymphoma 10	-0.06	0.817	0.23	0.390
Hs.405153	NOD1	Nucleotide-binding oligomerization domain containing 1	0.08	0.360	-0.19	0.035
Hs.200242	CARD6	Caspase recruitment domain family, member 6	0.28	0.344	**0.69**	0.134
Hs.446146	CARD8	Caspase recruitment domain family, member 8	nd	nd	0.33	0.287

Table S1. DIM-induced changes in expression of genes associated with apoptosis pathway in human CEM cells

			Length of treatment †			
			4 hours		24 hours	
**Unigene**	**Symbol** *	**Description**	**Log_2 R**	***p*-value**	**Log_2 R**	***p*-value**
Hs.38533	CRADD	CASP2 and RIPK1 domain containing adaptor with death domain	0.08	0.740	-0.07	0.541
Hs.513667	NOL3	Nucleolar protein 3 (apoptosis repressor with CARD domain)	0.18	0.423	0.55	0.078
Hs.499094	PYCARD	PYD and CARD domain containing	-0.01	0.910	0.01	0.869
Hs.103755	RIPK2	Receptor-interacting serine-threonine kinase 2	**0.66**	0.105	0.16	0.116
**CIDE domain family**						
Hs.249129	CIDEA	Cell death-inducing DFFA-like effector a	-0.16	0.822	**-1.91**	0.257
Hs.642693	CIDEB	Cell death-inducing DFFA-like effector b	-0.03	0.829	**-1.01**	0.093
Hs.484782	DFFA	DNA fragmentation factor, 45kDa, alpha polypeptide	-0.06	0.744	-0.32	0.206
**p53 and DNA damage response**						
Hs.431048	ABL1	C-abl oncogene 1, receptor tyrosine kinase	0.36	0.000	0.08	0.720
Hs.525622	AKT1	V-akt murine thymoma viral oncogene homolog 1	0.00	0.992	**-0.79**	0.095
Hs.80409	GADD45A	Growth arrest and DNA-damage-inducible, alpha	0.40	0.191	0.37	0.091
Hs.654481	TP53	Tumor protein p53	0.18	0.452	-0.16	0.448
Hs.523968	TP53BP2	Tumor protein p53 binding protein, 2	0.01	0.905	-0.23	0.176
Hs.697294	TP73	Tumor protein p73	0.06	0.938	-0.39	0.157
**Anti-apoptosis**						
Hs.550061	BRAF	V-raf murine sarcoma viral oncogene homolog B1	0.34	0.009	-0.15	0.161
Hs.435556	BFAR	Bifunctional apoptosis regulator	0.00	0.989	0.03	0.744
Hs.643120	IGF1R	Insulin-like growth factor 1 receptor	-0.28	0.248	0.01	0.921

Note: Genes are categorized according to functional groupings as outlined by the Human Apoptosis RT² Profiler™ PCR Array gene table; some genes belong to more than one category.

* Gene symbols in bold meet criteria for fold-change threshold and statistical significance.

† Log_2 fold-change (R) values are shown in bold if level of change is >1.5-fold (Log_2 R<-0.58 or >0.58) compared to vehicle (0.1% DMSO) control. *P*-values are compared to vehicle treatment as determined by a Student's *t*-test assuming equal variances. Genes highlighted in blue passed both fold-change and statistics criteria for at least one time-point.

‡ nd, not detected by RT^2 PCR profiler array at this time point (C_t >35).
