# Supplementary material for: 3,3′-Diindolylmethane Induces G1 Arrest and Apoptosis in Human Acute T-Cell Lymphoblastic Leukemia Cells
Source: PLoS One. 2012 Apr 13;7(4):e34975. doi: 10.1371/journal.pone.0034975 (PMC3325915; doi:10.1371/journal.pone.0034975)
Supplement: File S1 — Supplemental methods. Additional details on experimental methods are provided, including TUNEL analysis of CEM cells cultured in vitro, the CEM cell xenograft study and TUNEL analysis of human CEM cell xenografts. (PDF) [file pone.0034975.s003.pdf]

File S1. Supplemental methods

TUNEL analysis *in vitro*. The *In Situ* Cell Death Detection kit with Fluorocein (Roche Applied Science, Indianapolis, IN) was used to label DNA strand breaks and Guava Express Plus software (Millipore, Billerica, MA) was used to sort and quantify the amount of TdT incorporation. Briefly, cells were fixed in 1% paraformaldehyde (PFA) containing Triton X-100 on ice for 45 min. Cells were pelleted, rinsed, resuspended in ice cold 70% EtOH and stored at -20°C at least overnight to permeabilize prior to suspension in the TdT label/TdT enzyme mix for 1 hr at 37°C in the dark. Labeled cells were then rinsed with PBS, re-fixed in 4% PFA on ice for 20 min, resuspended in PBS and stored in the dark for up to one week at 4°C. Treatment of cells with 500 U DNase I produced a major population of high intensity TdT-fluorescein labeling and was used as a positive control for this assay, whereas a sample incubated with TdT label/no enzyme served as the negative control to gate background fluorescence. The percentage of TdT-fluorescein labeled cells was determined using the standard formula for the apoptotic index (AI), which was calculated as follows: AI = (number of TUNEL-positive cells/total number of cells) x 100.

These fixed and labeled samples were also visualized by fluorescence microscopy on a Zeiss Axiovert 200 fluorescent microscope. Stained samples were pelleted and resuspended in ProlongGold antifade reagent with DAPI (Invitrogen, Carlsbad, CA) and mounted between glass coverslips. Exposure settings were standardized using control samples before taking consecutive 20x magnification images of a single field with FITC and DAPI filters. These images were re-colored by filter channel and globally adjusted for brightness and contrast (when necessary) prior to merging into a single image file (ImageJ, National Institutes of Health).

CEM cell xenograft study. The xenograft experiment using human CEM cells was split into five cohorts in order to ensure consistent and adequate quantity and viability (>95%) of cells for injections. Each cohort consisted of four control mice and two mice for each treatment group. Mice within a cohort were randomized into six cages and fed control diet or diets containing 500 ppm I3C, 2000 ppm I3C or 100 ppm DIM (350 ppm BR-DIM) for one week prior to inoculation with CEM cells. All diets were provided to mice *ad libitum*; fresh food was provided daily, and diet consumption was monitored daily on a per cage basis. Two days prior to

inoculation, hair along the back of each mouse was removed with electric clippers to improve injection site visibility and measurement. Freshly collected CEM cells in a 1:1 (v/v) solution of medium/Matrigel were injected subcutaneously (*s.c.*) at two sites along the back of each mouse (anterior and posterior, 10^7 cells/site). Experimental diets continued for four weeks during xenograft growth. Starting on day 7 after inoculation, animal weights and nodule dimensions were assessed every third day with digital scale and digital calipers, respectively. Xenograft volumes were calculated using the equation for an ellipsoid ($L \times W^2 \times \pi/6$). During the first cohort of the experiment, we observed that anterior xenografts were difficult to palpate and were inconsistent compared to the same animal posterior site and within a given treatment group. The method of scruff restraint during *s.c.* injection likely resulted in inconsistent cell inoculations at the anterior position; thus, the anterior site was excluded from analyses for xenograft volume. Subsequent cohorts were similarly treated, though, to provide for consistency of experiment design. Four weeks following engraftment, mice were euthanized by CO_2 asphyxiation and necropsied. Blood was collected from the hepatic vein prior to excision of CEM cell xenografts and select tissues. The solid xenografts were weighed and divided into four quadrants of approximately equal volume and size, one of which was fixed in 10% neutral buffered formalin and processed for immunohistochemistry. The remaining xenograft fractions and tissues were immediately frozen in liquid nitrogen and stored at -80°C for future analysis.

TUNEL analysis of human CEM cell xenografts. Serial sections of xenografts were stained using the *In Situ* Cell Death Detection kit, POD (Roche) with the following modifications: 20 µg/ml Proteinase K for 15 min at 37°C and dilution of the POD converter solution 1:1 with buffer containing 1% BSA for 30 min at 37°C. Nova Red (Vector Laboratories; Burlingame, CA) was used as the chromagen, in place of DAB, and slides were counterstained with hematoxylin (Dako, Denmark). Multiple non-overlapping fields were imaged at 40x magnification for each section. At least 10 images per section, from a minimum of three sections per group, were analyzed with ImageJ software (≥30 images per treatment group), using the color deconvolution method of Ruifrok and Johnston [1]. Briefly, an initial image was used to determine the color vectors for the two stains, TUNEL positive (red), and TUNEL negative (blue). These values were added to the Color-Deconvolution

algorithm that was then run on each subsequent image to separate the colors into discrete images. The blue (negative) monochrome image was processed according to the MBF-ImageJ Manual for Particle Analysis, a multi-step process that results in the distinction of cellular boundaries to allow automatic counting by the software. Manual counting was used for determination of TUNEL-positive cells on all images (to eliminate false positive detection) and on a subset of blue monochrome images to validate the automatic counting process. The apoptotic-index (AI) was calculated as follows: AI = (manual count TUNEL positive/auto count negative) x 100.

1. Ruifrok AC, Johnston DA (2001) Quantification of histochemical staining by color deconvolution. Anal Quant Cytol Histol 23: 291-299.
